# Confederate States Hospital Reports

**Published:** 1864-05

**Authors:** 


### CONFEDERATE STATES HOSPITAL REPORTS.

*Report of wounded treated in Field Hospital of Hindman's Division, Army of Tennessee, after the battle of Chickamauga.* By CARLYLE TERRY, Chief Surgeon.

[We call especial attention to the valuable report of Surgeon Terry, and regret that we have not room for his accompanying remarks, of which, however, we present the following summary:

He first points out the great success of his operations, and compares them with his experience after Shiloh, where, with every advantage of position and appliances, he failed to reach such results. These cases were, many of them, treated in a stable, and most of them in rooms without fire. His success in such rooms was much greater than with those who *seemed to be* better situated. The patients were wet for days, and at all times exposed freely to wind and rain.

His only case of tetanus was No. 22, and he remarks that four cases after Shiloh were like this one, after amputation of arm, (which is not the experience of surgeons generally, the lower limbs seeming to be most liable to the disease.)

Chloroform was used in every instance without bad consequences.—ED.]

Case 1.—Marion Carlisle, second lieutenant company "G," 19th South Carolina; gun-shot wound; right knee-joint implicated, and femur comminuted; amputation middle third of thigh. Stump attacked with erysipelas about the fifth week, which caused much delay; finally recovered and was sent to General Hospital No. 16.

Case 2.—W. F. May, sergeant company "C," 10th South Carolina; wounded right knee, precisely like case 1. The amputation did well until the stump was nearly healed; but this case having been in a room with fire, five weeks after operation stump assumed a phlegmatic appearance, symptoms of pyemia appeared and he died October 16.

Case 3.—W. A. Gray, private company "K," 24th Alabama; compound fracture right thigh; amputation middle third, union by first intention, and discharged October 9.

Case 4.—J. B. Claridy, private company "C," 24th Alabama; wound of left knee joint; amputation lower third of thigh. Stump healed by first intention. Sent to the rear October 9.

Case 5.—John H. Dew, private company "A," 24th Alabama; compound comminuted fracture thigh; lay twenty-four hours on field. Amputation middle third forty-eight hours after wound was received. At first appeared to react but slowly, but was doing very well when sent to General Hospital, October 31.

Case 6.—W. F. Todd, private 10th South Carolina; compound comminuted fracture leg; amputation; condition good until 29th, when erysipelas supervened, which gave way to treatment. Sent to General Hospital, October 31, doing well.

Case 7.—A. B. Skipper, private company "A," 10th South Carolina; compound comminuted fracture; attempt to save the limb. After failing to arrest secondary hemorrhage (knee joint being found filled with pus,) amputation performed middle third thigh; general condition bad; no effort to rally; profuse suppuration supervened; secondary hemorrhage from sloughing of the artery. Died November 2.

Case 8.—W. W. Spivey, company "K," 22d Alabama; compound fracture thigh; amputation lower third; condition bad at time of operation owing to great loss of blood on the field; improved rapidly, and was sent to the rear, October 3, in a very promising condition.

Case 9.—Marion Barrett, corporal company "E," 19th Alabama; compound comminuted fracture; amputation lower third thigh; wound healing rapidly by granulation when sent to General Hospital, October 3.

Case 10.—H. W. Shilling, private company "A," 19th Alabama; compound comminuted fracture thigh; amputation middle third; condition bad; suffered much pain. Died of exhaustion, September 28.

Case 11.—J. W Powell, private company "A," 19th Alabama; compound comminuted fracture thigh; amputation lower third; condition good; stump granulating finely when sent away, October 3.

Case 12.—J. C. Minslett, private 39th Alabama; compound comminuted fracture thigh; amputation middle third; constitution bad; lost much blood on the field. Died September 30.

Case 13.—W. T. Kirby, private 50th Alabama; amputation middle third thigh; condition bad; stump did not do well; muscular and cutaneous tissues contracting very much, leaving bone protuding, which was again amputated, after which patient did very well, and was sent to General Hospital, October 13.

Case 14.—James Middleten, private company "L," 10th South Carolina; compound comminuted fracture leg; flap operation; recovered without any complication; sent to General Hospital, October 31.

Case 15.—Joshua Collins, private company "F," 10th South Carolina; same as above, but sent to General Hospital later, (November 22,) on account of not uniting so soon.

Case 16.—C. B. Foxworth, company "I," 10th South Carolina; compound comminuted fracture leg; amputation below the knee; progressing rapidly towards recovery, and sent to General Hospital, October 9.

Case 17.—W. Russell, corporal company "I," 28th Alabama; seat of wound not registered; amputation below the knee; nearly well and sent to General Hospital, September 30.

Case 18.—Robert Hatcher, private company "F," 24th Alabama; amputation arm; nearly healed and sent to General Hospital, October 3.

Case 19.—C. A. Pate, private company "C," 24th Alabama; gun-shot wound elbow joint; arm amputated; union by first intention; sent to General Hospital, October 3.

Case 20.—T. H. Lennard, private company "C," 24th Alabama; same as above; union by first intention; sent to General Hospital, October 3.

Case 21.—J. M. Smith, private 39th Alabama; amputation arm middle third; did not do well; had erysipelas for ten days, and wound not disposed to heal; left in the hands of the enemy November 26; will probably die.

Case 22.—W. P. Hunkerpillar, private company "B," 19th Alabama; wound elbow; amputation arm; patient did well until October 3, tetanic symptoms appeared, suppuration ceased, and he died October 7.

Case 23.—Ambrose New, sergeant company "I," 24th Alabama; resection elbow joint; very nearly well when sent to General Hospital, October 31.

Case 24.—John A. Jackson, private company "D," 28th Alabama; resection right shoulder joint; case did very well; wound filling up rapidly; October 30, the wound being nearly well and no suppuration, was sent to General Hospital.

Case 25.—W. F. Rodgers, private company "H," 28th Alabama; resection shoulder joint; did well, and was sent to General Hospital, October 30, in same condition as Jackson.

Case 26.—J. W. Clark, private company "A," 19th South Carolina; compound comminuted fracture thigh. This was one of the cases in the room with fire. This case progressed well towards bony union, as was shown by *post-mortem*. Pyemia developed on the 30th of September, and he died on the 8th of November.

Case 27.—William Kirkland, private company "A," 19th South Carolina; compound comminuted fracture thigh. Treated with double inclined plane; recovery complete, with only three inches shortening.

Case 28.—L. G. Williams, private company "C," 19th South Carolina; flesh wound of left thigh, and compound fracture same leg; treatment, fracture box; recovered.

Case 29.—George Little, private company "D," 19th South Carolina; compound comminuted fracture thigh; upper third; treatment, double inclined plane. This case was in the room with fire; pyemia made its appearance 29th September. Died October 5.

Case 30.—George Scott, sergeant company "D," 24th Alabama; compound comminuted fracture thigh; treatment, double inclined plane; recovery, bony union, and but slight discharge when sent to the rear, November 17.

Case 31.—Peter Chalons, company "G," 24th Alabama, aged 57; compound comminuted fracture thigh, very near the head of femur. No disposition to bony union; profused suppuration, and patient died of exhauston on 5th November.

Case 32.—F. Moore, private company "K," 24th Alabama; compound comminuted fracture left thigh; treatment, double inclined plane; united very rapidly, and was able to walk, November 15th, with crutches when sent to rear.

Case 33.—John Jenkins, private company "D," 28th Alabama; compound comminuted fracture thigh; same treatment; patient 45 years old; union perfect, and patient able to walk on crutches when sent away November 10.

Case 34.—J. Bishop, corporal company "D," 22d Alabama; ball passed through both hips, and bulbous portion urethra; bled profusely on the field; right femur fractured near its neck; suffered great pain for fifteen days; urine poured through wound, and repeated hemorrhages, from which he died October 8.

Case 35.—J. C. Archer, private company "D," 19th Alabama; compound comminuted fracture thigh; upper third; did remarkably well, and was sent to General Hospital October 20; treatment, double inclined plane.

Case 36.—J. M. Phelbs, private 22d Alabama; received two wounds; fracture neck of scapula and head of humerus, and compound fracture of thigh. No operation. Died of pyemia October 12.

Case 37.—Sydney Anderson, private 22d Alabama; compound comminuted fracture middle third thigh; double inclined plane; sent to General Hospital, doing very well, November 16; bony union.

Case 38.—Thomas Williams, private 39th Alabama; compound comminuted fracture thigh. No operation. Did not do well, and left in hands of enemy; will die.

Case 39.—Asa Johnson, private 39th Alabama; compound comminuted fracture thigh; treatment, double inclined plane; did well, and was sent to General Hospital November 16.

Case 40.—J. Winningham, private company "K," 10th South Carolina; wound of left lung; case progressed very well up to the 29th September, when he imprudently walked out and accidently fell down, when a severe hemorrhage supervened and patient slowly declined, and died October 10.

Case 41.—W. A. Green, private 25th Alabama; ball passed through right lung, entering just above right nipple; did remarkably well until October 3, then sent to General Hospital.

Case 42.—W. C. Pou, sergeant 10th Mississippi; ball passed through lung, upper lobe; about six days after had pneumonic symptoms, lasting five days; recovered.

Case 43.—James Morrison, private company "B," 19th South Carolina; wounded through chest and in fleshy part thigh; ball passed through lung; no hæmoptysis, and no constitutional disturbance, but rapid recovery; sent to rear October 10.

Case 44.—C. M. Turner, private campany "K," 19th South Carolina; wounded in lung; case made slow progress towards recovery, though suppuration was profuse until 17th October, when he died of hemorrhage.

Case 45.—Hugh Malony, private company "A," 24th Alabama; wound of left lung; recoved without any serious pulmonary disturbance; sent to General Hospital October 31.

Case 46.—H. T. Owens, private company "G," 22d Alabama; ball passed through right wing illium, coming out near left acetabulum; fecal matter escaped through the wound, and large quantity of blood poured through the bowels; never did well, and, at battle Missionary Ridge, was left in hands of enemy in dying condition.

Case 47.—E. F. Cooper, lieutenant company "G," 24th Alabama; wound of pelvis, ball entering centre of os innominata and not found; created but little disturbance and doing well; he was sent to General Hospital October 30.

Case 48.—W. Burke, private company "B," 24th Alabama; patient's health was much impaired; ball entered posterior aspect of thigh about midway, passing downwards along course of femoral vessels and came out near the knee. On the fourth day the wound assumed a gangrenous appearance throughout its whole extent, and the limb below became much swollen and with a livid hue, and also lost its temperature. Immediate amputation was resolved upon; the stump had a bad appearance. Death in twelve hours.

Case 49.—W. B. Brown, corporal company "F," 24th Alabama; ball passed through abdomen; passed fecal matter from both orifices for fifteen days; finally both healed, and feces passed naturally; no peritoneal symptoms; sent to rear, in safe condition, October 31.

